# The Possible Reduction Mechanism of Volatile Sulfur Compounds during Durian Wine Fermentation Verified in Modified Buffers

**DOI:** 10.3390/molecules23061456

**Published:** 2018-06-15

**Authors:** Yuyun Lu, Alicia Sarah Yoke Ling Fong, Jian-Yong Chua, Dejian Huang, Pin-Rou Lee, Shao-Quan Liu

**Affiliations:** 1 Food Science and Technology Program, Department of Chemistry, National University of Singapore, Science Drive 3, Singapore 117543, Singapore; yuyunlulyy@gmail.com (Y.L.); a0115026@u.nus.edu (A.S.Y.L.F.); chuajianyong@gmail.com (J.-Y.C.); chmhdj@nus.edu.sg (D.H.); 2 National University of Singapore (Suzhou) Research Institute, 377 Lin Quan Street, Suzhou Industrial Park, Suzhou 215123, China; 3 Shiro Corporation Pte Ltd, 1 Senoko Avenue, Singapore 758297, Singapore; Christine.Lee@shirocorp.com

**Keywords:** diethyl disulfide, ethanethiol, interconversion, yeast lees, mannoprotein

## Abstract

Durian fruit is rich in volatile sulfur compounds (VSCs), especially thiols and disulfides, which contribute to its onion-like odor. After fermentation, these VSCs were reduced to trace or undetectable levels in durian wine. The possible reduction mechanism of these VSCs (especially diethyl disulfide and ethanethiol) was investigated in a modified buffer in the presence of sulfite at different pH. An interconversion between diethyl disulfide and ethanethiol was found to be dependent on the pH: the higher the pH, the higher production of ethanethiol. It is suggested that, during durian wine fermentation, disulfides endogenous to durian pulp might be firstly converted into their corresponding thiols in the presence of reductant sulfite formed by yeast. The produced thiols as well as the thiols endogenous to the durian pulp were then removed by the mannoproteins of yeast lees.

## 1. Introduction

**Scheme 1:**
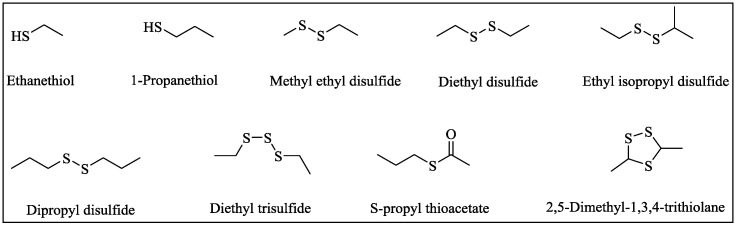
Structures of the main volatile sulfur compounds in durian fruit.

Durian is a well-known tropical fruit in Southeast Asia; this is largely a result of its particular onion-like odor which is contributed to by volatile sulfur compounds (VSCs), especially thiols (ethanethiol and propanethiol) and disulfides (diethyl disulfide and ethyl isopropyl disulfide) that are endogenous in durian pulp [1,2]. The major and common VSCs present in durian fruit are shown in Scheme 1. The VSCs extracted from durian pulp can react with glutathione with the release of hydrogen sulfide (H_2_S), which is reported to be beneficial to anticancer and cardiovascular diseases [3].

In addition, durian fruit is rich in carbohydrates, proteins, and fats relative to other fruits [2]; the fruit has also been found to be high in polyphenolic antioxidants, vitamins, dietary fibers, and minerals [1]. The consumption of durian fruit is beneficial to human health [4,5], but its co-consumption with alcohol has been reported to result in adverse effects such as palpitation and vomiting [6,7]. Similar symptoms have been known to occur in alcohol aversion therapy using disulfiram, which is a sulfur-containing drug with a disulfide bond [8].

Several studies have shown that VSCs which have been extracted from natural resources inhibit the activity of aldehyde dehydrogenase (ALDH), the key enzyme in alcohol metabolism with the ability to catalyze acetaldehyde to acetic acid [7,8,9]. The inhibition of ALDH is expected to result in an accumulation of acetaldehyde, which has been regarded as a cancer-causing substance during in vivo testing [9]. VSCs, especially diethyl disulfide, possess similar disulfide bond structures as disulfiram; they may inhibit ALDH activity by following the same inhibition mechanism [7,8].

Yeasts are the main factors that affect the metabolism of VSCs during wine fermentation [10,11,12]. Several new VSCs, such as 3-(ethylthio)-1-propanol, were produced via the metabolism of methionine in grape wine fermentation [13]. In addition, the production of H_2_S during yeast fermentation resulted in the accumulation of ethanethiol, *S*-ethyl thioacetate, and diethyl disulfide [14].

Previous studies have shown that volatile thiols are not stable; they were easily oxidized to disulfides [15,16,17] or formed non-volatile thiols through a reaction with polymeric phenols [18]. In addition, they can react with o-quinones to form thiol-substituted hydroquinones [19]. Furthermore, volatile thiols can also be removed by the cysteine residues of mannoprotein in yeast cell walls via the formation of a stable disulfide bond [20,21,22,23]. However, yeast lees reportedly adsorb both H_2_S and thiols but not sulfur, disulfur, thiophene, carbon disulfide, disulfides, or trisulfides [24].

On the other hand, disulfides can be reduced to their corresponding thiols by yeast under anaerobic conditions [25]. For example, diethyl disulfide was converted into ethanethiol in a modified buffer in the presence of sulfite; this interconversion was significantly affected by the pH of the buffer solution [26]. Previous studies have shown that sulfite is produced via oxidative pathways in aerobic degradation of ethanethiol [15] or as a result of the sulfate reduction sequence in yeasts during alcoholic fermentation [11].

Diethyl disulfide is one of the most abundant VSCs in durian fruit [2,27]. However, most of the VSCs, especially thiols and disulfides, in durian pulp are reduced to undetectable or trace levels by both *Saccharomyces* and non-*Saccharomyces* yeasts after durian wine fermentation [10,27,28,29]. The possible mechanism by which it is reduced has not yet been reported and is of particular interest to determine both the fate of VSCs and the mechanisms of such a transformation. The subsequent metabolism of the endogenous thiols (e.g., ethanethiol) and the thiols formed need to be further investigated and verified in terms of durian wine fermentation. Therefore, the objective of this study was to investigate the possible reduction mechanisms of VSCs, particularly thiols and disulfides, by yeast lees and mannoproteins during durian wine fermentation verified in modified buffers.

## 2. Results and Discussion

### 2.1. Changes of VSCs during Durian Wine Fermentation

**Figure 1:**
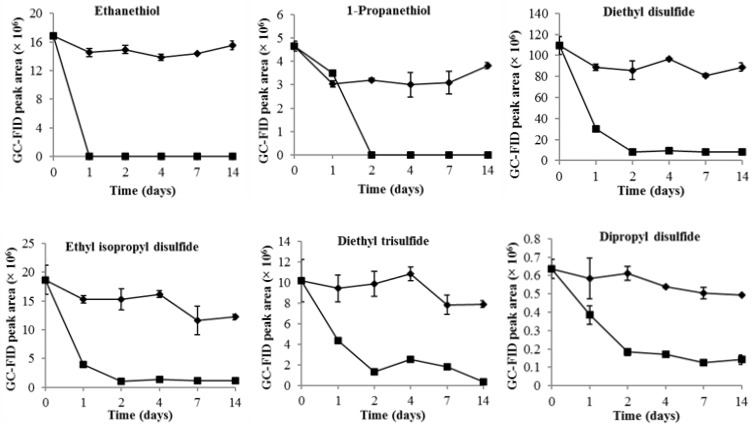
Changes of VSCs during durian wine fermentation and in durian pulp control. Durian wine fermentation (■), durian pulp control (◆).

The changes of VSCs during durian wine fermentation are shown in Figure 1. The volatile thiols (ethanethiol and 1-propanethiol) and sulfides (diethyl disulfide, ethyl isopropyl disulfide, diethyl trisulfide, and dipropyl disulfide) that were endogenous in durian pulp decreased rapidly to undetectable (thiols, FID (flame ionization detector) peak area < 2 × 10^5^) or trace levels (sulfides, FID peak area < 5 × 10^6^) by day 2 (Figure 1). On the basis of our previous studies [27,28,29], the odor activity value of VSCs, especially diethyl disulfide, remained above its threshold in durian wines, indicating that the unique durian odor was retained after fermentation. In contrast, these volatiles remained stable in the control groups, with only minor decreases that might have been caused by evaporation. This indicated that the significant reduction of VSCs during durian wine fermentation was closely related to the presence and the metabolism of yeasts. These results were consistent with previous studies [27,29]. In addition, another study reported a significant decline of diethyl trisulfide and dipropyl disulfide after spontaneous fermentation of durian pulp [30]; they suggested that the reduction was predominantly caused by the wild microorganisms. Furthermore, several studies found that sulfur compounds released during fermentation could also be removed by yeasts [21,24], which support the results of this study.

### 2.2. Changes of Total Thiols during Durian Wine Fermentation

**Figure 2:**
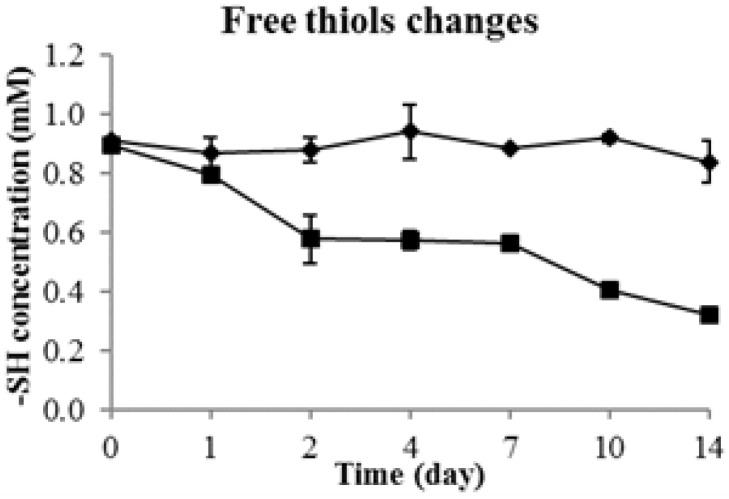
Kinetic changes of free thiols (determined by the 5,5′-dithiobis(2-nitrobenzoic acid), DTNB method) concentration during durian wine fermentation and in durian pulp control; durian wine fermentation (■), durian pulp control (◆).

The changes of total thiols in durian pulp (control group) and durian wine are shown in Figure 2. It was observed that the total free thiol concentration significantly decreased during durian wine fermentation; however, the control group remained essentially stable (Figure 2).

The results are consistent with the results of VSCs from the gas chromatograph-mass spectrometer (GC-MS) and flame ionization detector (FID) analysis (Figure 1). Nonetheless, the decrease in total thiol concentration was not as drastic as the GC results in which the volatile thiols sharply declined to undetectable levels by day 2. This could be explained by the presence of other non-volatile thiols (e.g., amino acids) in durian pulp such as glutathione and cysteine [31]. Durian fruit is a better source of essential amino acids and there is more than 0.7 g of cysteine per kilogram of fresh durian puree [2]. Therefore, the presence of sulfur-containing amino acids in durian pulp might have contributed to the total thiol concentrations in the 5,5′-dithiobis(2-nitrobenzoic acid) (DTNB) measurement.

### 2.3. Interconversion of Diethyl Disulfide and Ethanethiol

It has previously been shown that yeast lees are not able to consume disulfides [24]. Therefore, it is likely that the sulfides in durian pulp are converted into their corresponding thiols before being subsequently consumed by yeast during fermentation.

**Figure 3:**
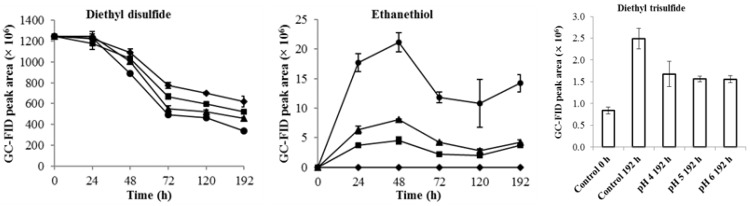
pH effect on the interconversion of diethyl disulfide and ethanethiol, control (◆), pH 4.0 (■), pH 5.0 (▲), pH 6.0 (●).

The interconversion of diethyl disulfide and ethanethiol under different pH conditions is shown in Figure 3. The results indicate that the interconversion rate and the production of ethanethiol were related to the pH value, with pH 6.0 producing the highest level of ethanethiol, which corresponds to the largest reduction of diethyl disulfide (Figure 3). On the other hand, the results also indicate that the reaction proceeded at pH 4.0, which is the pH value of durian wine fermentation; however, the interconversion rate was slower than that at high pH (Figure 3).

Diethyl disulfide in the control group also decreased throughout the treatment but no ethanethiol was produced (Figure 3). This might be because the diethyl disulfide had been oxidized to diethyl trisulfide (Figure 3) or had been lost through evaporation during the process. In addition, the lack of ethanethiol production in the control group indicated that the sulfite as the reducing agent was necessary for the interconversion of diethyl disulfide and ethanethiol (Figure 3). The production of ethanethiol was highest at 48 h in the presence of sulfite and then decreased (Figure 3). As the interconversion of diethyl disulfide and ethanethiol in the presence of sulfite is reversible, this may indicate that an equilibrium level of diethyl disulfide and ethanethiol was reached [26]. Therefore, the reduction of ethanethiol could be ascribed to the dimerization to diethyl disulfide to achieve equilibrium and, subsequently, the reproduction of the ethanethiol (Figure 3).

Disulfides, especially diethyl disulfide and ethyl isopropyl disulfide, are abundant in durian pulp (Figure 1); the sulfite (SO_3_^2−^) can be produced by yeasts during fermentation through oxidative pathways in aerobic degradation of ethanethiol [15] or from the sulfate reduction sequence in yeasts [11]. On the basis of these results, it is likely that diethyl disulfide was converted into ethanethiol. Therefore, if the produced thiols were removed by yeasts during fermentation, the conversion would follow Le Chatelier’s principle [32], resulting in a continuous reaction with the production of thiols from disulfides or trisulfides, even under low pH conditions.

### 2.4. Consumption of Thiols by Yeast Lees

**Figure 4:**
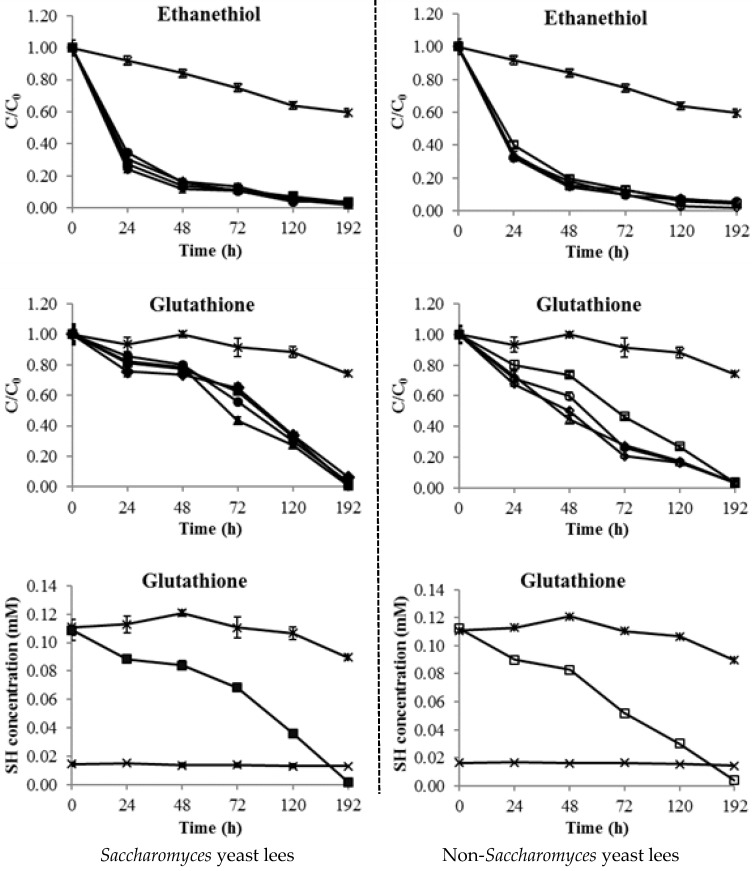
Thiols (ethanethiol and glutathione) consumption by eight types of yeast lees. Thiol control (ж), *S. cerevisiae* EC-1118 (■), *S. cerevisiae* Merit (▲), *S. cerevisiae* R2 (●), *S. cerevisiae* 71B (◆), *T. delbrueckii* Biodiva (□), *W. saturnus* NCYC22 (Δ), *L. thermotolerans* Concerto (○), *T. delbrueckii* Prelude (◊), yeast lees control (×).

The kinetic changes of free thiols (ethanethiol and glutathione) caused by yeast lees (4 *Saccharomyces* and 4 non-*Saccharomyces* yeast lees) are shown in Figure 4. Both ethanethiol (volatile thiol) and glutathione (non-volatile thiol) significantly decreased to trace levels (over 90% reduction) after the addition of yeast lees (Figure 4). The control groups of ethanethiol and glutathione only showed a slight decrease (~20% reduction); this may have been partially caused by evaporation (ethanethiol) because of its lower boiling point [33] and/or might have been oxidized to the corresponding disulfides [15,16,17]. In addition, all yeast lees did not result in the reduction of diethyl disulfide by following the same processing procedures (data not shown), indicating that yeast lees are unable to consume disulfides [24].

Notably, the reduction of ethanethiol was much faster than that of glutathione in all yeast lees treatments. Over 60% of ethanethiol was reduced in the first 24 h; in contrast, the glutathione only reduced by around 20% (Figure 4). The results indicate that the reduction of ethanethiol was more easily caused by the yeast lees as compared to glutathione. Because glutathione is a significantly larger molecule than ethanethiol, this may suggest that the reduction of thiols was via an attachment mechanism in which the compounds occupied space on the exterior of the yeast cell walls. Accordingly, smaller molecules could attach onto the yeast cell walls per unit time because of a weaker degree of steric hindrance [34].

A previous study showed that yeast lees could completely consume methanethiol after incubation for 190 h in a modified tartrate buffer [23]. The reduction of thiols might have been caused by the cysteine residues on yeast cell wall mannoproteins [35], which may have bound the added free thiols to form a disulfide bond [22]. On the other hand, the final total thiol concentration in samples (e.g., the addition of glutathione and *S. cerevisiae* EC-1118/*T. delbrueckii* Biodiva yeast lees) decreased to levels lower than that of the yeast lees control (Figure 4), indicating the decline of the thiol units of cysteine residues on yeast cell wall mannoproteins. Overall, these results indicate that the decreases of both ethanethiol and glutathione were mainly caused by the addition of yeast lees.

### 2.5. Consumption of Thiols by Mannoprotein

**Figure 5:**
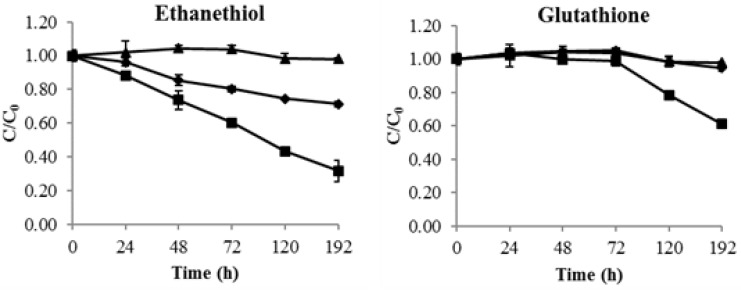
Thiols (ethanethiol and glutathione) consumption by mannoproteins, thiol control (◆), thiol + mannoprotein (■), mannoprotein control (▲).

To verify that the reduction of thiols was caused by cell wall mannoproteins of yeast lees, commercial mannoproteins were used instead of yeast lees. The results showed that both the ethanethiol and glutathione decreased gradually when mannoproteins were added (Figure 5); however, diethyl disulfide did not decrease (data not shown). The slight decrease of ethanethiol in the control group might be ascribed to evaporation due to its low boiling point or its oxidization to the corresponding disulfides, as discussed above. On the other hand, the glutathione control remained constant; however, it decreased in the samples containing the mannoproteins. This confirmed that the decrease of thiols could mainly be attributed to the addition of mannoproteins.

**Scheme 2:**
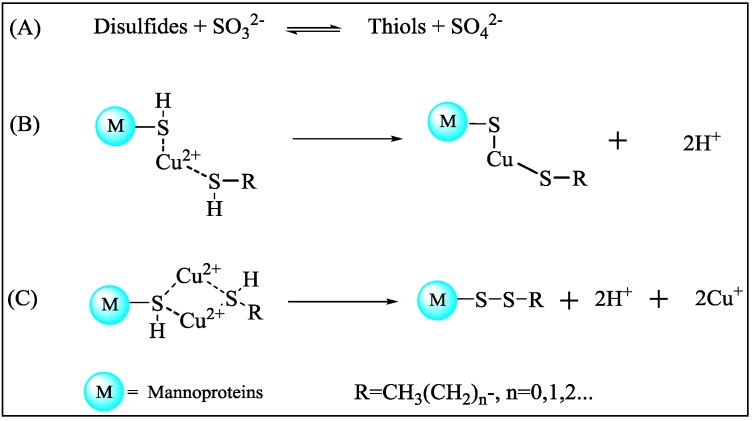
Proposed interconversion mechanisms of disulfides caused by yeast fermentation. (**A**) The interconversion of disulfides and thiols in the presence of sulfite; (**B**) The thiols bound to mannoproteins with copper insertion within disulfide bridges; (**C**) The thiols bound to the mannoproteins after Cu^2+^ ions were reduced and liberated as Cu(I) ions.

Therefore, it is possible that disulfides endogenous to durian pulp might be firstly converted into their corresponding thiols by sulfite generated by yeasts during durian wine fermentation (Scheme 2A). These thiols could bind to some metallic cations [23], such as copper ions (Cu(II)), found in the cell wall mannoproteins by forming stable complexes, thus preventing the liberation of H_2_S and thiols. Two possible mechanisms of thiols reduction were previously proposed on the basis of the oxidation mediated by Cu(II) [20]. The thiols could bind to the mannoproteins with copper insertion within disulfide bridges (Scheme 2B). In addition, yeast cell wall mannoproteins, Cu(II), and free thiols could form an intermediate binding. Subsequently, the Cu^2+^ ions were reduced and liberated as Cu(I) ions (Scheme 2C) while the thiols became bound to the mannoproteins [20].

## 3. Materials and Methods

### 3.1. Durian Wine Fermentation with Saccharomyces Cerevisiae EC-1118

Durian (D666) imported from Malaysia was purchased from a local supermarket in Singapore. The durian flesh and seeds were manually separated, and the flesh was blended with deionized water at a ratio of 3:7 (*w*/*w*) to form a homogeneous mixture. The pH and °Brix of the durian pulp was adjusted to 4.0 (1 M dl-malic acid) and 20 (sucrose), respectively. Pasteurization was carried out at 60 °C for 20 min and the effectiveness of pasteurization was confirmed by spread plating on potato dextrose agar (PDA).

The pure culture of *S. cerevisiae* EC-1118 was prepared as reported previously [28], and the pre-culture was prepared by inoculating 5% (*v*/*v*) of *S. cerevisiae* EC-1118 into the pasteurized durian pulp, which was then incubated at 25 °C for 48 h with the colony forming units (CFU) of over 107 per milliliter. The pre-culture (1%, *v*/*v*) was inoculated into the pasteurized durian pulp (300 mL) in 500-mL sterile Erlenmeyer flasks. A control group without inoculation of pre-culture was also prepared. Triplicate experiments were carried out and the fermentation was conducted at 25 °C for 14 days statically. The samples were taken periodically to monitor the changes of VSCs.

### 3.2. Interconversion of Diethyl Disulfide and Ethanethiol in Modified Buffer

The interconversion of diethyl disulfide and ethanethiol was investigated to determine the possibility of their conversion under fermentation conditions. The procedures followed a reported method with minor modification [26]. Triplicate reactions were carried out in 500-mL blue cap bottles wrapped with aluminum foil to prevent photooxidation. Diethyl disulfide (100 mg/L) and sodium sulfite (Na_2_SO_3_, 1.5 mM) were added into the different buffers (KHPh/HCl, pH 4.0; KHPh/NaOH, 5.0 and KH_2_PO_4_/NaOH, 6.0). Diethyl disulfide without sodium sulfite was added to the corresponding buffers as the control groups. All reactions were conducted at 25 °C for 192 h and the samples were taken periodically to monitor the changes of diethyl disulfide and ethanethiol using headspace solid-phase microextraction (HS-SPME) with a carboxen/poly (dimethylsiloxane) fiber (85 µm coating; Supelco/Sigma-Aldrich, Santa Clara, CA, USA).

### 3.3. Detection of Volatile Sulfur Compounds

Analysis was performed using a GC-MS/FID. All samples were adjusted to pH 2.5 with 1 M HCl to cease fermentation, and a 5-mL aliquot was extracted at 60 °C for 50 min by HS-SPME under a rotational speed of 250 rpm. The SPME fibre was desorbed at 250 °C for 3 min in the injection port of an Agilent 7890A gas chromatograph coupled to an Agilent 5675C triple-axis MS and FID. An Agilent DB-FFAP capillary column (60 m × 0.25 mm i.d., Agilent, Santa Clara, CA, USA) coated with a 0.25-mm thick film of polyethylene glycol, modified with nitroterephthalic acid was used to separate the volatile compounds. Helium at a flow rate of 1.2 mL/min was used. The temperature was programmed from 50 °C (5 min) to 230 °C (30 min) at a rate of 5 °C/min. The eluate was then passed into the FID and MS for analysis. The ionization was produced at 70 eV electron impact at 230 °C and acquisition mode was full scan (2.78 scan/s) from 3 to 71 min between 25 and 550 atomic mass units (amu).

### 3.4. Consumption of Thiols by Yeast Lees

Yeast lees were obtained after alcoholic fermentation in grape musts (grape variety: Scarlet royal) inoculated with four *Saccharomyces* (*S. cerevisiae* EC-1118, *S. cerevisiae* R2, *S. cerevisiae* 71B, *S. cerevisiae* MERIT.ferm,) and four non-*Saccharomyces* yeasts (*Torulaspora delbrueckii* Biodiva, *T. delbrueckii* Prelude, *Lachancea* (previously *Kluyveromyces*) *thermotolerans* Concerto, and *Williopsis saturnus* var. *saturnus* NCYC 22) at 25 °C for 10 days, respectively. The yeast lees were obtained after alcoholic fermentation of grape must instead of from durian wine fermentation and were used to avoid the possible results effects. The yeast lees were washed with deionized water 10 times to remove potassium hydrogen tartrate and residual alcohols to avoid organoleptic variations [23]. Subsequently, the yeast lees were freeze dried for three days and stored at −20 °C before use.

One volatile thiol (ethanethiol) and one non-volatile thiol (glutathione) were used to investigate the free thiols consumption capacity of yeast lees in modified buffer. Ethanethiol (1 mM) and glutathione (0.1 mM) were added into 40 mL of KHPh/HCl buffer (pH 4.0) containing each of the eight yeast lees (0.1 g), respectively. The buffers which only contained the respective yeast lees and the respective thiols were the corresponding control. All treatments were protected from light and conducted at 25 °C for 192 h in static condition. The samples (2 mL) were taken at 0, 24, 48, 72, 120, and 192 h and were centrifuged immediately to remove the solid yeast lees. The supernatants were used to measure the free thiol concentration using 5,5′-dithiobis(2-nitrobenzoic acid) (DTNB) at 412 nm [33].

### 3.5. Consumption of Thiols by Mannoproteins

Ethanethiol (1 mM) and glutathione (0.1 mM) were added into 40 mL of KHPh/HCl buffer (pH 4.0) containing 0.1 g of mannoproteins (OptiRed^®^, Lallemand, Pickering, QC, Canada). The buffers with the addition of ethanethiol or glutathione without mannoproteins were the pure thiols control. The buffer with addition of mannoproteins without thiols was the mannoproteins control. All treatments were protected from light and conducted at 25 °C for 192 h under static condition. The samples (2 mL) were taken at 0, 24, 48, 72, 120, and 192 h and were centrifuged immediately to remove the solid mannoproteins. The supernatants were used to measure the free thiol concentration using DTNB at 412 nm [33].

### 3.6. Quantification of Free Thiol

The free thiol concentration was measured using DTNB as a reagent to produce a yellow-colored thiolate anion [33]. A sample solution (0.2 mL) was added into 0.1 M sodium phosphate buffer (1.0 mL, pH 6.8) before adding the DNTB solution (0.3 mL, 1mM). The mixture was vortexed for 10 s and allowed to stand for 10 min at room temperature. The absorbance was measured at 412 nm in a UV/VIS spectrophotometer (Shimadzu, Kyoto, Japan), and the free thiol concentrations of ethanethiol and glutathione were calculated based on their respective standard curves under the same condition.

### 3.7. Statistical Analysis

The ANOVA test using the software SPSS^®^ 17.0 for Windows^®^ (SPSS Inc., Chicago, IL, USA) was used to analyze the data. Results were considered significant if *p* < 0.05. Average values and standard deviations were calculated from the triplicate experiments.

## 4. Conclusions

VSCs, especially thiols and sulfides, originally present in the durian pulp significantly decreased to low or trace levels after alcoholic fermentation. Disulfides, such as diethyl disulfide, were unable to be directly reduced by the yeast cells. A possible mechanism of their removal would be via their reduction to their respective thiols, such as ethanethiol. The thiols were then able to bind to cysteine residues on yeast cell wall mannoproteins, which resulted in the removal of the VSCs from durian pulp during fermentation.
